# The Jekyll and Hyde of Cellular Senescence in Cancer

**DOI:** 10.3390/cells10020208

**Published:** 2021-01-21

**Authors:** Dilara Demirci, Bengisu Dayanc, Fatma Aybuke Mazi, Serif Senturk

**Affiliations:** 1 Izmir Biomedicine and Genome Center, Izmir 35340, Turkey; dilara.demirci@ibg.edu.tr (D.D.); bengisu.dayanc@msfr.ibg.edu.tr (B.D.); fatmaaybuke.mazi@msfr.ibg.edu.tr (F.A.M.); 2 Izmir International Biomedicine and Genome Institute, Dokuz Eylul University, Izmir 35340, Turkey

**Keywords:** cancer, cellular senescence, SASP, therapy-induced senescence, senostatic, senolytic

## Abstract

Cellular senescence is a state of stable cell cycle arrest that can be triggered in response to various insults and is characterized by distinct morphological hallmarks, gene expression profiles, and the senescence-associated secretory phenotype (SASP). Importantly, cellular senescence is a key component of normal physiology with tumor suppressive functions. In the last few decades, novel cancer treatment strategies exploiting pro-senescence therapies have attracted considerable interest. Recent insight, however, suggests that therapy-induced senescence (TIS) elicits cell-autonomous and non-cell-autonomous implications that potentially entail detrimental consequences, reflecting the Jekyll and Hyde nature of cancer cell senescence. In essence, the undesirable manifestations that generally culminate in inflammation, cancer stemness, senescence reversal, therapy resistance, and disease recurrence are dictated by the persistent accumulation of senescent cells and the SASP. Thus, mitigating these pro-tumorigenic effects by eliminating these cells or inhibiting their SASP production holds great promise for developing innovative therapeutic strategies. In this review, we describe the fundamental aspects and dynamics of cancer cell senescence and summarize the comprehensive research on the adverse outcomes of TIS. Furthermore, we underline the rationale and motivation of emerging senotherapeutic modalities surrounding the removal of senescent cells and the SASP to help maximize the overall efficacy of cancer therapies.

## 1. Introduction

Almost 60 years ago, Hayflick and Moorhead challenged Carrel’s original proposition that normal cells have an infinite replication capacity. On that account, Hayflick performed a series of experiments with diploid primary cells derived from various human embryonic tissues. These studies unveiled the fact that normal cells propagated in culture can replicate for a limited and probably predetermined number of generations, after which they undergo an irreversible arrest of cell growth, thus disproving Carrel’s theory of cellular immortality [1,2]. This phenomenon is now known as the Hayflick limit or, as it will be called herein, replicative senescence [3,4,5,6]. Over the course of six decades, cellular senescence has been established as an adaptive stress response mechanism in physiological and pathological processes with both beneficial and detrimental consequences for human health [7,8,9,10]. 

Depending on the cell type and conditions, different subtypes of cellular senescence such as DNA-damage-induced senescence, stress-induced senescence (SIS), and oncogene-induced senescence (OIS) have been defined [11,12,13]. Earlier studies have shown that cellular senescence program is a key component of embryonic development and tissue remodeling and may potentially function as a tumor suppressor mechanism against carcinogenesis [9,14,15]. Work in recent decades have debated the longstanding fundamental paradigm of senescence irreversibility. In striking contrast to the traditional definition, these research efforts have provided mounting evidence that this complex phenotype is not a static, permanent, and docile state, but rather entails a constantly evolving multi-step process with cell-autonomous and non-cell-autonomous implications and often deleterious effects on tissue homeostasis. In the context of cancer therapy, this capacity is primarily due to the fact that senescent cells remain viable and bioactive for long periods of time and eventually resume proliferation while emitting heterotypic signals to their microenvironment [16,17,18,19].

The century-old classic novel “The Strange Case of Dr. Jekyll and Mr. Hyde” by Robert Louis Stevenson explores the duality of human nature—specifically, the natural existence of a dual personality, good and evil, in the same individual. Arguably, the balance between good and evil is what makes us human. In some sense, this theme is analogous to the dual nature of cellular senescence, where it can be both beneficial (Jekyll) and detrimental (Hyde). Our objective in this review is to synthesize the recent scientific advances pertaining to the duality of cellular senescence, with a heightened interest in cancer, and to present scientific advances and challenges in exploiting this phenotype in cancer therapies. With this motivation, we first revisit the hallmarks of cellular senescence primarily by stressing the morphological and molecular biomarkers, as well as the regulation and functions of the senescence-associated secretory phenotype (SASP). The key effector mechanisms and different subtypes of cellular senescence are then briefly summarized. Next, we discuss the biological significance of cellular senescence in normal physiology and shift the focus to cancer, referencing the good and evil natures of this phenomenon. From there, we elaborate on the concepts of therapy-induced tumor cell senescence, stemness, and senescence escape. Finally, we accentuate the impact and the rationale of emerging senotherapeutic approaches surrounding the targeting of senescent cells and the SASP to help develop novel cancer treatments.

## 2. The Hallmarks and Molecular Mechanisms of Cellular Senescence

### 2.1. Morphological and Molecular Biomarkers of Senescent Cells

**Figure 1:**
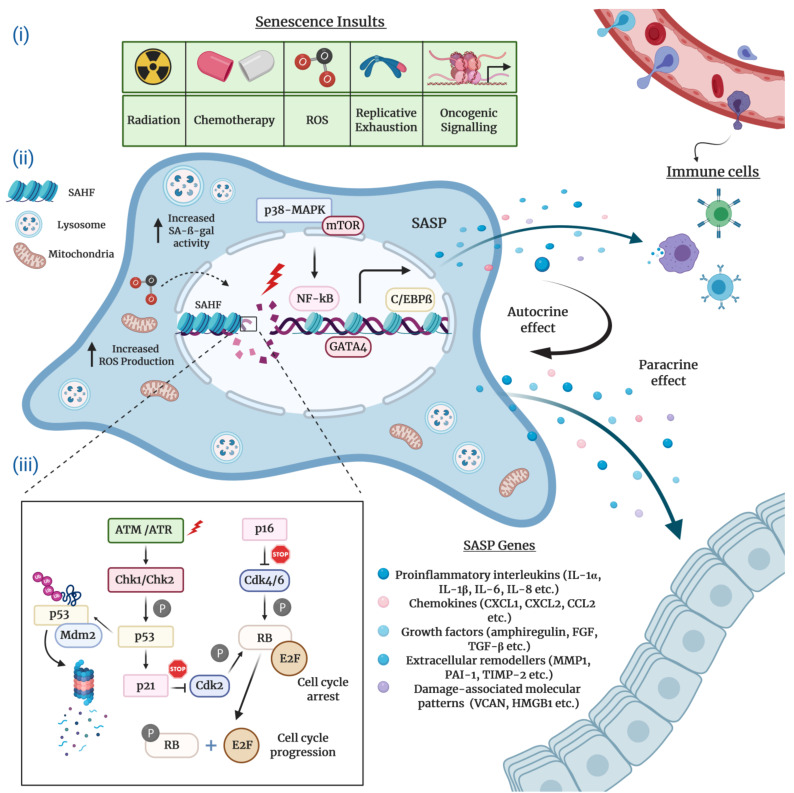
The hallmarks and molecular mechanisms of cellular senescence. The figure summarizes 3 major attributes of cellular senescence. (**i**) Intrinsic and extrinsic insults causing senescence: The extrinsic factors causing senescence-related cell cycle arrest comprise of radiation and chemotherapy, whereas intrinsic factors harbor increased reactive oxygen species (ROS) accumulation, aberrant oncogene activation, and replicative exhaustion. (**ii**) Molecular hallmarks of senescent cells and pathways regulating senescence-associated secretory phenotype (SASP) production and immune cell infiltration (recruitment of immune cells to the SASP-rich milieu): SASP expression is predominantly controlled by the p38-MAPK and mTOR pathways and C/EBPβ, GATA4, NF-κB transcription factors. Senescent cells enriched for SASPs disseminate a wide assortment of senescence cues (proinflammatory interleukins, chemokines, growth factors, extracellular remodelers, damage-associated molecular patterns/DAMPs) to the surrounding cells (paracrine effect). At the same time, these cues influence on the senescent cell itself (autocrine effect). Clearance of senescent cells is actualized via immune surveillance mechanisms, and (**iii**) molecular mechanisms modulating cell cycle arrest: DNA-damage dependent and DNA-damage independent mechanisms regulate the key effector mechanisms p53/p21^Cip1^ and pRb/p16^Ink4a^ to initiate and maintain cellular senescence.

Typically characterized by the inability to replicate their DNA and cellular growth arrest, cultured senescent cells exhibit a series of distinct morphological and chemical hallmarks which distinguish them from proliferating cells. Perhaps the most notable molecular markers are multiple or enlarged nuclei and flattened cytoplasm, an increased number of lysosomes and Golgi apparatus, elevated pH-dependent senescence-associated β-galactosidase activity (SA-β-gal), and resistance to apoptosis [16,20,21]. Senescent cells are also frequently characterized by impaired nuclear integrity; the formation of persistent nuclear DNA damage foci and DNA-damage response (DDR); deregulated metabolism; protein and lipid damage; global epigenetic changes in their chromatin landscape; the formation of senescence-associated heterochromatin foci (SAHF); and, of course telomere attrition, the hallmark of replicative senescence [12,13,22,23,24] (Figure 1).

### 2.2. The Senescence-Associated Secretory Phenotype (SASP) of Senescent Cells

A striking feature of virtually all senescent cells is the widespread changes in protein expression that involve a specific signature for secreted molecules, collectively known as the SASP. The SASP consists of a myriad of biologically active soluble and insoluble factors which can be grouped into the following major categories: proinflammatory interleukins and chemokines; growth factors; extracellular matrix proteins and remodeling enzymes; damage-associated molecular patterns; and extracellular vehicles greatly enriched for enzymes, miRNAs, and DNA fragments [25,26,27,28,29,30]. Recently, Basisty et al. developed a comprehensive and quantitative proteomic atlas that can potentially serve as a reference and guide for the identification of novel soluble (sSASP) and exosome/extracellular vesicle SASP (eSASP) factors. The atlas is currently limited to two distinct cell lines induced to senesce by various stress factors. However, the authors expect the resource to be continuously updated by depositing new SASP profiles derived from different cell types and senescence-inducing conditions [30]. In essence, the abundance and heterogeneous composition of the SASP is context-dependent, partly explaining how the SASP can exert profoundly diverse and sometimes contradictory functions in numerous biological processes such as tissue remodeling, inflammation, and age-related pathologies including cancer [31]. 

The SASP regulation in senescent cells has been the subject of numerous studies. The findings collected in these studies substantiate the notion that the SASP production is coordinated by a complex network of signaling cascades that involve to a large extent transcriptional but also post-transcriptional mechanisms (Figure 1). Stress-inducible kinase p38 mitogen-activated protein kinase (p38-MAPK), mammalian target of rapamycin (mTOR), cytosolic DNA-sensing cyclic GMP–AMP synthase (cGAS)–stimulator of interferon genes (STING), the Ataxia telangiectasia mutated (ATM)/ATM- and RAD-3 related (ATR)-activated IκB kinase (IKK)/NEMO complex, and the GATA binding protein 4 (GATA4) axis constitute the most prominent upstream regulators of the pro-inflammatory senescence phenotype [31,32,33,34,35,36]. Upon stimulation by stress conditions, these interactive signaling pathways converge towards the activation of a transcriptional program managed by the nuclear factor kappa B (NF-κB) and the CCAAT-enhancer binding protein β (C/EBPβ), the core effectors that initiate and maintain SASP gene expression [31,37,38]. A number of studies suggest that the Janus kinase–signal transducer and activator of transcription (JAK/STAT) and NOTCH pathways also play a crucial role in the transcriptional regulation of SASP components through C/EBPβ [39,40,41]. 

The DDR signaling pathway is a critical mediator of the SASP. Available research indicates that the direct activation of ATM/ATR protein kinases in response to persistent DNA damage inhibits the autophagic degradation of GATA4, which, in turn, activates NF-κB to initiate and maintain the SASP network [42,43]. Differently, several reports describe the DNA damage-independent control of the SASP induction, which, in general, involves the p38-MAPK-mediated activation of NF-κB [32,44,45,46,47]. Intriguingly, mitochondrial dysfunction-associated senescence (MiDAS) is a distinct form of DDR-independent cellular senescence wherein the cells undergoing MiDAS display a unique SASP profile dictated by AMP-activated protein kinase (AMPK)-mediated p53 activation [47]. 

Epigenetic mechanisms are also pronounced in the modulation of cellular senescence and SASP constituents. For example, the histone variants macroH2A1 and H2AJ accumulate in human primary lung fibroblasts during OIS and play an important role in the positive and negative regulation of SASP production [48,49]. Similarly, epigenetic modifiers including lysine methyltransferase 2A (KMT2A, also known as MLL1), high-mobility group B protein 1 and 2 (HMGB1 and HMGB2), and bromodomain-containing protein 4 (BRD4) modulate the senescence secretome by orchestrating the chromatin landscape around the SASP gene loci [50,51,52,53]. Moreover, the downregulation of sirtuin 1 (*SIRT1*) gene and the enhancer of zeste 2 polycomb repressive complex 2 subunit (*EZH2*) gene in senescent cells positively regulate SASP factors, which are mediated by transcriptomic changes through the post-translational modifications of histones [46,54]. In addition to transcriptional mechanisms, the expression of SASP genes is regulated at the post-transcriptional level. In particular, MAPK-activated protein kinase 2 (MK2), a downstream effector of the p38-MAPK and mTOR pathways, modulates the mRNA stability of a subset of SASP components by means of ARE-mediated decay [55,56].

### 2.3. Molecular Mechanisms Underlying Cellular Senescence 

The complex network of molecular events that execute cellular senescence has been extensively reviewed elsewhere [40,57,58]. Nonetheless, for the sake of the completeness and consistency of this review, we will mention the critical effector pathways. Many lines of research convincingly attest that the onset and maintenance of permanent senescence arrest is controlled by the p53/p21^Cip1^ and the retinoblastoma protein (pRb)/p16^Ink4a^ tumor suppressor pathways (Figure 1). In principle, the activation of either one or both of these crucial pathways can readily induce cellular senescence. Notably, genetic mutations or epigenetic silencing of these pathways obliterates the senescence response in most cell types, occasionally paving the way for cancer initiation and progression [59]. 

Mechanistically, in its active hypophosphorylated form the pRb binds to and sequesters E2F family of transcription factors and induces growth arrest in the G1 phase of the cell cycle. To achieve this, pRb suppresses the transcription of several E2F target genes encoding a repertoire of essential proteins indispensable for DNA replication and cell cycle, thus blocking the subsequent entry into and progression through the S phase. The regulation of cellular senescence by E2F is often correlated with context-dependent local or global structural epigenetic modifications such as chromatin remodeling and SAHF formation. Consistent with this, the promoters of E2F target genes are enriched for repressive histone modifications (mainly H3K9me3 and H3K27me3) which result in gene expression changes that eventually contribute to the regulation of cellular senescence. Upon phosphorylation by Cyclin D and Cyclin-dependent kinase 4 and 6 (CDK4 and CDK6), a complex that is negatively regulated by the p16^Ink4a^ tumor suppressor protein, pRb switches to an inactive state and releases E2F, thus stimulating cell cycle progression [60,61,62,63]. 

The p53 transcription factor, the guardian of the genome integrity, plays a pivotal role in the induction and maintenance of cellular senescence. Following exposure to genotoxic or non-genotoxic stress, p53 gets activated and promotes cell cycle arrest via DDR-dependent and DDR-independent mechanisms. The specific activity of p53 is tightly controlled by virtue of positive and negative regulators and post-translational modifications. In response to stress stimuli, p53 is phosphorylated and stabilized by ATM/ATR and Checkpoint kinase 1 and 2 (Chk1/2) protein kinases, releasing it from MDM2, an E3 ubiquitin ligase that negatively regulates p53 via ubiquitination and proteasomal degradation. Once activated, p53 selectively increases the transcription of various target genes, in particular p21^Cip1^ (*CDKN1A*), a potent CDK inhibitor which executes the p53-mediated control of cellular senescence. The p21^Cip1^ protein binds to and inhibits the activity of Cyclin E/CDK2 and Cyclin D/CDK4 complexes, thus activating pRb and blocking cell cycle progression [17,64,65,66]. Finally, yet importantly, depending on the cellular identity and stress factors, antitumor mechanisms coordinated by the p53 and pRb pathways may engage different subtypes of cellular senescence.

## 3. The Significance of Cellular Senescence: From Homeostasis to Cancer

Almost a century ago, Muller and McClintock postulated that telomeres, the specialized structures found at the ends of linear eukaryotic chromosomes, were critically essential for the maintenance of chromosomal stability [67,68]. Later, in the 1970s, Olovnikov and Watson speculated independently that the chromosomal ends become shorter with each round of DNA replication [69,70]. Landmark discoveries in the following years have revealed that telomeres protect the chromosomes against degradation and interchromosomal fusions, thus contributing to the maintenance of genome integrity [71,72,73]. Furthermore, telomeres control the number of successful divisions that a normal cell can undergo before entering permanent growth arrest, the state of replicative senescence. This phenomenon is explained by progressive shortening of telomeres due to decreased or lack of telomerase reverse transcriptase enzyme (TERT) expression or activity [74,75]. Accordingly, the reintroduction of *TERT* gene into somatic cells promotes bypass of replicative senescence [76,77]. Unlike somatic cells, certain stem cell populations, germ cells, and rapidly dividing cells including cancer cells retain a high telomerase activity. Convincingly, the maintenance of telomere homeostasis via sustained or restored telomerase activity is associated with immortality and thereby considered a hallmark of cancer [78]. Notably, *TERT* gene amplification, translocations to euchromatic regions, and highly recurrent promoter mutations are the most common mechanisms of telomerase reactivation in cancer [79,80,81,82,83].

Stress-induced senescence (SIS), also known as stress-induced premature senescence, is a global spectrum of acutely evoked growth arrest programs. Functionally, SIS protects the organism from the potentially harmful effects of excessive accumulation of damaged cells in tissues and serves as a cell-intrinsic barrier against preneoplastic transformation. While sharing similar molecular and functional features with replicative senescence, SIS is essentially distinct in the nature of provoking stimuli. Almost any form of cell extrinsic or cell intrinsic stressors other than telomere dysfunction/damage can potentially induce SIS. Triggers include oncogene activation or suppression of tumor suppressor genes, cytokines, mitochondrial dysfunction, reactive oxygen species (ROS), DNA damage or nucleotide depletion [84,85,86,87,88]. Spindle stress or nucleolar stress, unfolded protein response and endoplasmic reticulum (ER)-stress, and metabolic and epigenetic alterations are the other well-recognized SIS-inducing stimuli [16,89,90]. On the whole, these stress factors directly or indirectly cause damage to biological macromolecules such as nucleic acids, carbohydrates, lipids, and proteins. 

Cellular senescence can be divided into two fundamentally different categories according to the functionality and kinetics of the senescence process, a priori the extent and duration of the stimulus [9,91]. Acute (transient) cellular senescence, mainly caused by cell-extrinsic factors, often targets a defined group of cells and is generally acknowledged as a beneficial and tightly regulated physiological process in development and tissue injury repair [92,93]. Importantly, acute senescent cells can orchestrate their self-recognition and immune-mediated clearance through effector mechanisms manifested by the SASP factors, further implying that this scheduled or programmed process is temporal [33,94]. By contrast, chronic (persistent) cellular senescence is a non-programmed process with no specific target cell population and has detrimental effects on tissue homeostasis. In many settings, chronic cellular senescence is associated with prolonged exposure to genotoxic stress and progressive macromolecular damage to cellular components [9,95]. There are studies which detail that chronic cellular senescence evolves from an acute state, especially when the immune clearance is severely impaired. The vast majority of these reports suggest that the subsequent accumulation of persistent senescent cells amid the secretion of multi-faceted SASP factors is usually harmful and can both aggravate and contribute to age-associated pathologies, including atherosclerosis, renal pathologies, and cancer [96,97,98]. Taken together, these seemingly contradictory beneficial and detrimental functions make this vital molecular process a double-edged sword with both opportunities and obstacles for therapeutic targeting.

## 4. The Implications of Therapy-Induced Senescence in Cancer

Traditional cancer treatments have relied on genotoxic and cytotoxic therapies such as chemotherapy and radiation therapy or an effective combination of both. These therapies typically provide high enough doses of drugs to induce complete cell death in rapidly dividing cancer cells [99]. Under such circumstances, cytotoxic strategies also cause significant toxicity to normal cells, leading to severe side effects in multiple organ systems [100]. Despite the fact that these interventions deliver therapeutic benefits and overall improvement in survival outcomes, tumors frequently develop resistance and advance to more aggressive primary or metastatic diseases [101,102,103]. When compared with cytotoxic therapies, cytostatic therapies do not exert direct cytotoxicity but rather aim to slow down or stop the growth of tumor cells [104,105]. On the basis of the antitumor activities of senescence process in early-stage premalignant lesions, senescence-inducing strategies have been valued as alternative therapies in the battle against cancer. An effective and promising strategy to induce cytostasis in cancer treatment is therapy-induced senescence (TIS). Aside from their cytotoxic actions, when administered in low doses or intermittent regimens certain conventional therapies display cytostatic activity and promote TIS in human cancer tissues [105,106,107]. 

Cisplatin and doxorubicin are the first genotoxic stressors shown to trigger cancer cell senescence [108,109]. Similarly, topoisomerase inhibitors, antimetabolites, alkylating agents, and microtubule inhibitors have been reported to induce TIS [110,111,112]. Interestingly, targeted therapies can also provoke cellular senescence in cancer cells. Research on targeted pro-senescence therapies is specifically concentrated on the reactivation of tumor suppressor pathways (e.g., p53-MDM2 and p53-p21 axis) or the therapeutic targeting of oncoproteins (e.g., Myc), the inhibition of cell cycle machinery (e.g., CDC7, CDK4/6, and PARP), the suppression of cellular pro-survival pathways including receptor tyrosine kinases and their downstream effectors (e.g., PI3K/Akt/mTOR, PTEN, and aurora kinase B), casein kinase 2 (CK2), and epigenetic modulators [113,114,115,116,117]. 

Given these efforts, TIS is considered as a powerful intervention in conventional cancer therapy. However, the strategies exploiting pro-senescence therapies are complicated, the reason being that TIS can be transient and reversible in nature. More importantly, the persistent accumulation of senescent cancer cells and the SASP following TIS are endowed with cell-autonomous and non-cell-autonomous mechanisms which facilitate senescence escape, invasiveness, therapy resistance, and cancer recurrence [118,119]. The pioneering study by Elmore et al. discovered that breast cancer cells, acutely exposed to doxorubicin, could evade the stress insult and produce resistant clones that were no longer responsive to senescence-inducing cytotoxic drugs, including the doxorubicin rechallenge. Interestingly, senescence-resistant cells had normal intracellular drug accumulation and functional DDR machinery, and the senescence evasion was coupled to the elevated expression of proliferative cell cycle regulators, in particular Cdc2/CDK1 [120,121,122]. Similarly, Roberson et al. found that p53-null and p16^Ink4a^-deficient human lung carcinoma cells escape from TIS through the increased expression and phosphorylation of Survivin, a downstream effector of Cdc2/CDK1 survival signal. Moreover, the inhibition of Cdc2 and Survivin activity increases chemotherapy efficiency and reduces tumor recurrence [123]. Another study by Yang et al. revealed that the intermittent administration of chemotherapeutic compounds generates aggressive cell variants that acquire the ability to evade cellular senescence. To that end, lung tumor cells undergoing doxorubicin-induced senescence were then exposed to other stress-inducing cytotoxic agents. Interestingly, cells subjected to sustained selective pressure spontaneously reentered cell cycle and eventually produced senescence revertants with enhanced potential for migration and invasion. Finally, the revertants outcompeted parental counterparts in tumor growth when implanted subcutaneously into nude mice, concluding that aggressive cell variants may emerge as an outcome of chemotherapy [124]. Similarly, the work by Saleh et al. utilized topoisomerase II inhibitors to trigger cellular senescence in lung, colon, and breast cancer cell lines. In support of the previous findings, the live cell tracking of the senescence phenotype with special reporters identified reemerging clones that recovered from cellular senescence and acquired proliferative and tumorigenic potential, strongly suggesting an escape from or reversal of TIS [119].

Recently, a breakthrough study has unveiled a new, yet unexpected, cell-intrinsic relationship between spontaneous escape from TIS and senescence-associated stemness (SAS). This discovery has definitely advanced our understanding on the plasticity of senescent cancer cells and the significance of attacking these cells in cancer therapies. To that end, the authors initially compared the gene expression profiles of doxorubicin-exposed versus untreated lymphomas, specifically senescence-competent primary Eμ-*Myc* transgenic *Bcl2*-overexpressing lymphomas (dubbed as Eμ-*Myc*;*Bcl2*) and senescence-incompetent Suv39h1-deficient Eμ-*Myc*;*Bcl2* lymphomas. Transcriptome data uncovered that the key signaling components of TIS essentially overlap with stemness pathways. Importantly, senescence-competent cells were enriched for the adult tissue stem-cell gene signature, suggesting that senescent cancer cells acquire phenotypic and functional features of stem cells. Furthermore, the authors found that turning off the expression of Suv39h1 or p53, two critical effectors of senescence, using a tamoxifen-inducible system results in cell cycle progression. Strikingly, the senescence-released lymphoma cells with SAS capacity display markedly higher tumor initiation potential when compared to never senescent lymphomas. Mechanistically, SAS reprogramming in post-senescent cancer cells was strongly attributed to epigenetic mechanisms that enhanced cell-intrinsic Wnt signaling, largely excluding the potential role of non-cell-autonomous mechanisms [125,126]. Together, this study illustrates that TIS can trigger a cell-autonomous and senescence-associated stemness reprogramming in proliferation-arrested cancer cells and those cells that manage to escape from senescence evolve into more aggressive tumor-initiating cells. 

Today, there is strong research evidence in support of the notion that the escape from cellular senescence in cancer represents, in principle, a natural phenomenon of reversibility that is not limited to TIS but can occur with other forms of senescence insults. A number of studies have demonstrated that arrested cells can readily escape from OIS by mechanisms linked to *TERT* derepression/reactivation and the inactivation of p53 and pRb pathways [127,128,129]. Furthermore, unpublished observations from our group suggest that hepatocellular carcinoma (HCC) cells retain the potential to regain proliferative capacity following TGF-β−mediated prolonged senescence. 

## 5. Therapeutic Targeting of Senescent Cells and the SASP in Cancer

As detailed here and elsewhere, the negative implications associated with the pro-senescence cancer therapies are generally attributed to the accumulation of senescent cancer cells and their SASP composition and intensity [130,131]. In view of that, the number of studies focusing on counterbalancing the potential detrimental effects of such therapies has experienced a significant expansion in recent years. In particular, the regulation and therapeutic targeting of the senescence phenotype and the SASP has become an area of extensive research. Currently, the gold-standard therapeutic approaches to keep these cells under control are (i) boosting immune surveillance mechanisms, (ii) intervening the SASP production and activity through senomorphics, and (iii) the selective elimination of senescent cells with senolytic agents [132,133,134,135]. We note that the immune-mediated clearance of senescent cells is beyond the coverage of this review, thus we would like to divert the reader to other reports to comprehend this topic [31,94,136].

### 5.1. SASP Activity in Cancer and Anti-SASP Therapies

One potential mechanism by which senescent cancer cells display both anti- and pro-tumorigenic activities is the Jekyll and Hyde dynamics of the SASP network. From the anti-tumorigenic perspective, the SASP factors may reinforce the cell-intrinsic control and maintenance of the senescence fate and instruct the paracrine transmission of secondary senescence to SASP-receiving premalignant cells. In addition, the non-cell-autonomous SASP can engage immunosurveillance mechanisms and ensure that senescent cancer cells are eliminated from the tumor tissue [26]. Yet, in some contexts, the accumulation of senescent cancer cells, again by virtue of the SASP, is strongly implicated in promoting aggressive cancer cell behaviors and immunoediting [137,138,139,140]. Therefore, attenuating the constituents or the regulators/effectors of the SASP, without actually compromising their tumor suppressive functions, embodies a fundamental therapeutic advantage in cancer. On this account, early studies proposed that the SASP can be successfully suppressed by calorie-restricting diets, the activators of telomerase and sirtuin family of proteins, broad anti-inflammatory agents (e.g., glucocorticoids) as well as the activators of autophagy [141,142,143,144,145]. In contrast, recent studies have encouraged exploiting anti-SASP approaches for more specific targets. 

**Figure 2:**
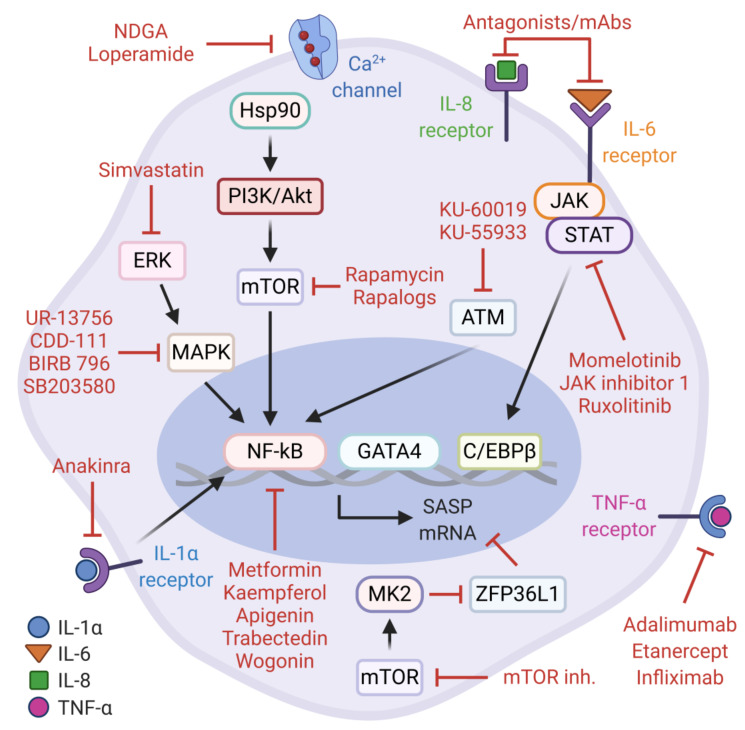
Anti-SASP therapies. Senostatics suppress markers of senescence and blunt SASP production. The main targets include NF-κB and C/EBPβ transcription factors and their upstream signaling networks. These types of senostatics provide the cell-intrinsic repression of the SASP. Autocrine and paracrine effects of the SASP are prevented by targeting the main components of the SASP, especially IL-1α, IL-6, IL-8, and TNF-α.

As alluded to earlier, the secretory component of senescent cells is mainly orchestrated by NF-κB and C/EBPβ. These transcription factors are modulated by upstream signaling networks which clearly represent attractive therapeutic targets for senostatic interventions. Senostatics are characterized as the drugs that repress markers or phenotypes of senescent cells without promoting apoptotic cell death. To date, several natural or pharmacological agents have been proposed to effectively blunt these pathways and mitigate the deleterious consequences of pro-tumorigenic SASP factors (Figure 2).

One of these druggable mechanisms is the PI3K/Akt/mTOR pathway. The physiological inhibition of this signaling cascade by rapamycin, a pharmacological mTOR inhibitor, contributes to longevity and delays age-related pathologies in various model organisms [146,147,148,149]. In the context of cellular senescence, rapamycin and rapalogs (rapamycin analogs) can attenuate the mTOR-dependent transcriptional activity of NF-κB and alleviate the synthesis of pro-tumorigenic SASP factors without preventing the senescence arrest. These observations are explained by the translational inhibition and subsequent reduction in cell-surface bound IL-1α, which can suppress the IL-1α/NF-κB positive feedback loop [150]. Consistent with this, Herranz et al. also reported that rapamycin and other mTOR inhibitors (Torin 1 and NVP-BEZ235) can inhibit OIS-mediated SASP gene expression based on the observations that mTOR signaling controls the stability of SASP transcripts via MK2-mediated negative regulation of ZFP36L1, a zinc-finger RNA-binding protein with mRNA decay activity. Furthermore, rapamycin administration in mice with transposon-mediated N-RAS^G12V^ expression in hepatocytes demonstrates potent activity against pro-inflammatory SASP during liver cancer initiation [56]. Together, these findings may likely explain the beneficial effects of rapamycin in age-related pathologies and extend its therapeutic merit to intervene the pro-tumorigenic SASP. Similarly, the pharmacological perturbation of the p38-MAPK and MK2 signaling cascade with selective inhibitors (e.g., CDD-111, SB203580, UR-13756, and BIRB 796) results in the significant suppression of pro-inflammatory SASP production in replicative senescent cells, which warrants further investigation on inhibiting this axis in age-related pathologies, including cancer [33,55,151]. 

As noted earlier, the DDR kinases ATM and ATR can impact on the SASP by activating the NF-κB transcription factor via the upstream regulator, GATA4. In agreement with this, the inhibition of the ATM/NF-κB signaling axis by potent small molecule compounds, KU-60019 and KU-55933, suppresses markers of cellular senescence and the SASP, emphasizing the senotherapeutic value of these agents [152,153,154]. Another level of repression against pro-tumorigenic activities of senescent cancer cells can be achieved by trabectedin, an alkylating agent derived from *Ecteinascidia turbinata*. Notably, trabectedin modulates the NF-κB pathway and reduces SASP gene expression in doxorubicin-induced senescent cancer cells resulting in the sensitization to Fas-mediated apoptosis and the inhibition of TIS escape mechanisms [155]. 

Metformin, an antidiabetic/anti-aging medicine in clinical use for over six decades, also exerts well-established pleiotropic effects towards the inhibition of cancer-promoting signaling pathways [156,157,158]. Furthermore, metformin is experimentally validated to exert senostatic activity and attenuate the increased burden of senescent cells in many contexts [159,160,161,162]. For example, during H-RAS^G12V^-induced senescence in human lung fibroblasts, metformin interferes with the activation of the IKK pathway, sparing p38-MAPK, and inhibits the nuclear translocation of NF-κB, leading to the suppression of the SASP [162]. In a targeted therapy context, metformin can synergistically enhance the in vitro and in vivo antiproliferative effects of CDK4/6 inhibition in experimental models of head and neck squamous cell carcinoma (HNSCC). In this setting, metformin blocks both the mTOR signaling pathway and the senescence-associated reprogramming of cancer stemness induced by CDK4/6 inhibitor, a known Jekyll and Hyde of CDK4/6 inhibition in cancer treatment [160]. Thus, metformin can be potentially repositioned as a senostatic agent, alone or in combination with other drugs, in relevant clinical settings for cancer treatment. 

Interestingly, many naturally occurring flavonoids including kaempferol, apigenin and wogonin have substantial capacity to effectively suppress cellular senescence and the SASP [163,164]. The report by Perrott et al. describes that apigenin downregulates the expression and secretion of several SASP components in senescent human fibroblasts. Although the precise molecular mechanism remains unclear, the findings suggest that apigenin can strongly inhibit the p38-MAPK and NF-κB pathways. Moreover, the secretome of apigenin-treated senescent fibroblasts, as opposed to untreated controls, fails to induce an aggressive phenotype in breast cancer cells [165]. In accordance with these findings, another study found that wogonin and kaempferol can inhibit NF-κB activity via IRAK1/IκBα signaling cascade in DNA damage-induced senescent fibroblasts [166]. Collectively, evidence from these studies merits future investigation of naturally occurring flavones and broad-spectrum anti-inflammatory senostatics to evaluate their translational applicability in cancer cell senescence.

The JAK/STAT pathway plays an important role in chronic sterile inflammation, a hallmark of aging and age-related diseases [167]. Moreover, to a great extent, the accumulation of senescent cells and the SASP contributes to this process [27]. To infer a causal link between the JAK/STAT signaling and the SASP, Xu et al. performed a series of experiments in human primary preadipocytes and human umbilical vein endothelial cells (HUVECs), and aged mice. As anticipated, the inhibition of JAK/STAT pathway by potent JAK1/2-specific inhibitors including JAK inhibitor 1, momelotinib, and ruxolitinib alleviated the SASP in irradiation-induced senescent cells. Further, ruxolitinib decreased both systemic and adipose tissue inflammation and increased physical activity in frail mice [168]. Consistent with these findings, ruxolitinib treatment rescues truncated lamin A (progerin)-induced cellular senescence and the SASP in cultured MRC-5 cells and the Hutchinson–Gilford progeria syndrome (HGPS)-derived fibroblasts. In addition, ruxolitinib administration delays premature aging phenotypes in murine model of progeria [169]. Together, the JAK/STAT inhibition can signify a senostatic venue in the context of cancer cell senescence. 

Simvastatin, an HMG-CoA reductase inhibitor with anti-hyperlipidemic activity, can reduce inflammatory responses by inhibiting the isoprenylation of Rho-family of GTPases [170]. By the same token, simvastatin can suppress the expression of several SASP components in irradiation-induced senescent human fibroblasts, without actually affecting the proliferation arrest and SA-β-gal activity. More importantly, simvastatin mitigates non-cell-autonomous effects of pro-tumorigenic SASP components by inhibiting paracrine activation of the ERK pathway in breast cancer cells, providing a rationale to explore simvastatin as an anti-SASP agent in cancer therapies [171]. Notably, Ca^2+^ channel inhibitors, loperamide and nordihydroguaiaretic acid (NDGA), have been recently identified to have senomorphic activity in DNA repair-deficient *Ercc1^−/−^* mouse embryonic fibroblasts [16,172].

Just as importantly, several therapeutic drugs currently available in clinical use for autoimmune and autoinflammatory diseases such as rheumatoid arthritis can directly target the SASP components or their receptors. In this context, the vast majority of the relevant studies focus on targeting IL-1 Receptor (anakinra), IL-6 Receptor (tocilizumab, siltuximab), IL-6 (sirukumab), and TNF-α (adalimumab, etanercept and infliximab) that have an immense potential to be effectively repositioned as precision senostatics to block the detrimental outcomes of the SASP [31,173,174,175,176]. 

In summary, scientific evidence collected through these studies suggest that senostatic agents may potentiate cancer therapies by modulating or inhibiting the proinflammatory SASP components. However, the context-dependent complexity and intensity of the SASP network, and the non-senescence related functions of the SASP factors may be critical limitations to an effective senostatic intervention. Therefore, careful consideration must be given when applying the senostatic agents and future studies will need to focus on addressing these challenges.

### 5.2. Senolytic Therapies

**Figure 3:**
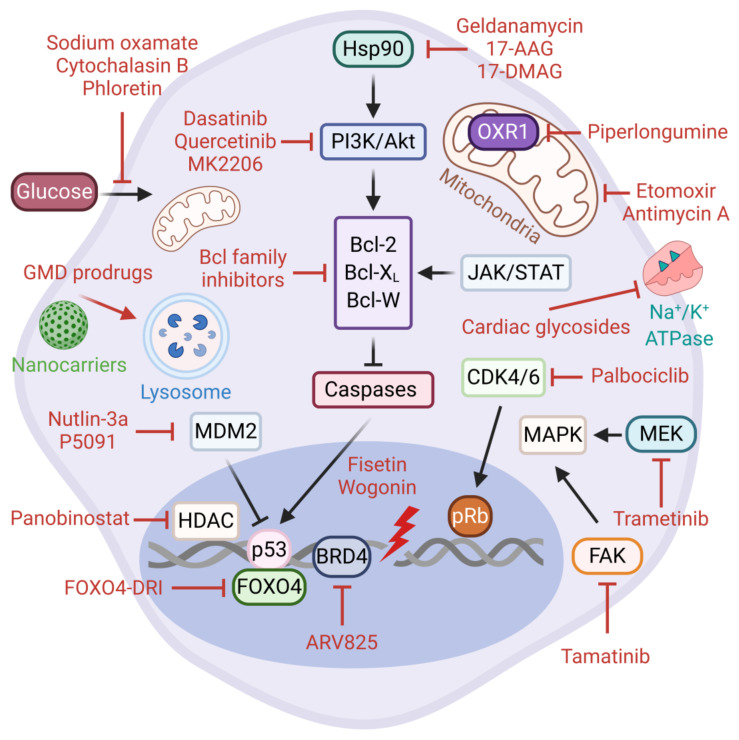
Senolytic therapies. Senolytics mainly target the Senescent Cell Anti-apoptotic Pathways (SCAPs). The SCAPs are activated to protect senescent cells from apoptosis triggered by any form of pro-apoptotic insults, including the SASP. The most common SCAPs are Hsp90, Bcl-2 family proteins, p53, and the PI3K/Akt/mTOR pathway. Several senolytic agents identified to date are depicted. Directed targeting of senescent cells is achieved by the nanoparticle-based delivery of senolytics.

As opposed to normal proliferating cells, senescent cells are highly resilient to cell intrinsic and extrinsic apoptotic stimuli. The Senescent Cell Anti-apoptotic Pathways (SCAPs) are the key molecular players involved in protecting senescent cells from pro-apoptotic insults, including their own SASP factors [177]. These pro-survival pathways are deemed druggable vulnerabilities and to some extent they represent the Achilles’ heel of senescent cells. The SCAPs identified to date include Bcl-2 family of anti-apoptotic proteins (e.g., Bcl-2, Bcl-X_L_, Bcl-W, Mcl-1), p53-p21 axis, hypoxia-inducible factor 1-alpha (HIF-1α), heat shock protein 90 (Hsp90), several receptor tyrosine kinases, and the PI3K/Akt/mTOR pathway (Figure 3).

The targeting of SCAPs via senolytic therapies aims to selectively eliminate senescent cells that accumulate in tissues during aging and age-associated pathologies without impacting healthy cells [178,179,180]. The first senolytic agents were discovered through hypothesis-driven bioinformatics research. These are dasatinib, a dual BCR/ABL and Src family tyrosine kinase inhibitor, and quercetin, a plant flavonoid with potent inhibitory activity against Bcl-2 family members, HIF-1α, and receptor tyrosine kinases including the PI3K/Akt/mTOR signaling pathway. Notably, the senolytic activities of dasatinib and quercetin are cell-type specific, however they display an increased efficacy and range of target cells when used in combination [181,182,183,184,185]. 

Over the years, new senolytic drugs with a broader spectrum and higher specificity have been discovered. For example, Chang et al. exploited the cell-based phenotypic screening of a chemical compound library and identified a small molecule BH3 mimetic ABT-263 (navitoclax) as an inhibitor of Bcl-2, Bcl-W, and Bcl-X_L_ [186]. Navitoclax is a broad-spectrum senolytic which exhibits cell type-independent activity in experimental models of in vivo senescence [187,188]. Similarly, ABT-737, an analogue of navitoclax, is senolytic against partial hepatectomy-induced senescent hepatocytes, as well as lung and epidermal senescent cells in irradiated mice [189,190]. Despite their robust senolytic activity, the existing Bcl-2-targeting inhibitors are unfortunately associated with severe hematological toxicity, prompting the identification of better senolytics with less side effects. Notably, the innovative PROTAC technology is emerging as a powerful strategy to reduce the platelet toxicity of navitoclax [191]. On the same premise, Zhu et al. investigated the senolytic potential of fisetin, yet another example of a natural flavonoid, as well as the two selective Bcl-X_L_ inhibitors, A1155463 and A1331852. As expected, these compounds induced apoptosis in senescent HUVECs and human fibroblasts but with less toxicity than navitoclax [192]. Importantly, dietary fisetin can reduce cell viability in several cancer cell lines of breast, colon, lung, HCC, pancreatic, prostate, bladder, and glioma origin [193,194]. Similarly, when co-administered with fisetin, wogonin induces apoptosis in HCC through the activation of the caspase 3 pathway and the accumulation of p53 [195]. Tamatinib (R406), a potent ATP-competitive (Type I) Syk inhibitor, has recently been identified as a novel senolytic in senescent human dermal fibroblasts (HDFs). The senolytic effects of tamatinib were strongly associated with the inhibition of cell survival pathways through the reduced phosphorylation of both focal adhesion kinase (FAK) and p38-MAPK. Interestingly, navitoclax can further potentiate the senolytic activity of tamatinib, suggesting that, alone or in combination with other drugs, tamatinib represents a good candidate for further investigation [196]. In conclusion, the elimination of senescent cells by the inhibition of the central anti-apoptotic factors and their upstream regulatory pathways represents an effective strategy for targeting cancer cell senescence.

Hsp90 is a highly abundant molecular chaperone that plays a critical role in the folding and stabilization of client proteins involved in diverse cellular processes, including cell cycle control, apoptosis, and signal transduction. On this premise, a number of natural or synthetic Hsp90 inhibitors such as geldanamycin and geldanamycin derivatives (17-DMAG and 17-AAG) display senolytic properties in multiple cell types in vitro and animal models in vivo [172,197]. In general, Hsp90 inhibitors pleiotropically target and abrogate NF-κB and PI3K/Akt/mTOR survival pathways [198]. Consistent with this knowledge, the inactivation of Akt signaling by an allosteric inhibitor, MK2206, can also trigger apoptosis in prostate cancer cells senesced by an androgen antagonist enzalutamide [199]. Piperlongumine, a natural extract with anti-tumor activities in non-small cell lung cancer (NSCLC), is a senolytic in various contexts [57,113,117,200,201]. Piperlongumine-induced senolysis is associated with increased ROS production via the degradation of antioxidant protein oxidation resistant 1 (OXR1) and the inhibition of the PI3K/Akt/mTOR pathway [202,203,204]. Overall, the inhibition of Hsp90 and the client pro-survival pathways can provide robust senolytic activity in certain types of cancer. Panobinostat, an FDA-approved potent inhibitor of histone deacetylase with antineoplastic or cytotoxic activity, can selectively eliminate persistent senescent preneoplastic cells that accumulate following chemotherapy-treated NSCLC and HNSCC cell lines [205]. These findings strongly support the evaluation of Panobinostat as a post-chemotherapy senolytic in appropriate clinical settings.

The p53/p21^Cip1^ axis has also also been evaluated in the context of senolytic interventions. Recent reports have identified Nutlin-3a, a highly specific MDM2 antagonist, and P5091, a ubiquitin specific ligand 7 (USP7) inhibitor, as potent senotherapeutics. These agents promote MDM2 ubiquitination and degradation and the reciprocal accumulation of p53 [206,207]. The forkhead box O (FOXO) transcription factors regulate many cellular processes, including cell cycle progression and apoptosis [208,209]. The interaction between p53 and FOXO4 at the sites of DNA damage contributes to cellular senescence [210]. Recently, a rationally designed D-retro-inverso (DRI) isoform of FOXO4 (FOXO4-DRI) peptide has been shown to block this interaction and promote the nuclear exclusion of p53, which eventually results in the Caspase-3/7-mediated apoptosis of senescent cells. Furthermore, FOXO4-DRI can effectively eliminate doxorubicin-induced senescent cells both in vitro and in vivo and counteract the negative effects of chemotherapy, representing a valuable senolytic opportunity in cancer therapy [211].

Increased glucose and lipid metabolism can support survival in senescent cancer cells in the absence of other growth stimuli. Accordingly, the inhibition of glucose metabolism via phloretin, sodium oxamate, or cytochalasin B showed a senolytic effect in therapy-induced senescent lymphomas due to the high dependency of senescent cells on hypermetabolism. From the same perspective, the etomoxir-mediated suppression of fatty acid oxidation as well as the inhibition of oxidative phosphorylation by antimycin A exert a senolytic effect in senescent lymphomas [212]. These results would suggest that hypermetabolism could be potentially exploited as a therapeutic vulnerability in TIS tumors. Furthermore, a novel study by Wakita et al. unveiled the fact that the inhibition of BRD4 activity by BET inhibitor ARV825 is an effective senolytic mechanism through autophagy-activated apoptosis in doxorubicin-induced senescent colorectal cancer cells [213]. This study raises avenues for future investigation—in particular, the potential contribution of autophagy machinery to other senolysis mechanisms.

Another inspiring senolysis approach is based on cardiac glycosides (CGs), which are synthetic or plant-derived steroid-like compounds that selectively inhibit the Na^+^/K^+^-ATPase found on the plasma membrane [214]. CGs are traditionally used to treat atrial fibrillation and cardiac failure [215]. Using a chemical library screening of FDA/EMA-approved drugs and plant extracts, Triana-Martinez et al. identified several CGs, including proscillaridin A, ouabain, and digoxin with strong in vitro senolytic effects on lung cancer and melanoma models, independent of the senescence insult. The same study then tested the senolytic potential of digoxin (a drug in clinical use) on chemotherapy-treated subcutaneous lung tumors and patient-derived xenografts of breast cancer. The combination of digoxin with clinical senogenic anticancer agents eradicated senescent cancer cells and significantly reduced tumor volume and senescent cell markers [216]. Consistent with this, ouabain has been validated to eliminate bystander senescent cells in clinically relevant in vitro and in vivo models of oncogene- and therapy-induced senescence, further contributing to the drug discovery of a broad-spectrum senolytic arsenal [217].

In conclusion, senescent cancer cells can evade apoptosis through the activation of anti-apoptotic and pro-survival mechanisms (collectively, the SCAP network), which potentially enables them to resist self-destruction. Therefore, the inhibition of SCAP-related molecules individually or the combinatorial targeting of multiple components across the SCAP network can result in selective apoptosis, highlighting the clinical utility of these strategies. Although many of the currently available senolytic drugs have certain drawbacks, those with strong preclinical data and limited off-target toxicities, in particular, are likely to translate into clinical research in the near future.

### 5.3. Directed Targeting of Senescent Cells

Although senotherapies are decisively formulated to execute selective cytotoxic action in senescent cells, there are often side effects inherent to the course of delivery and implementation. In recent years, novel tools and versatile systems have been developed to directly deliver and release therapeutic compounds into senescent cells. One of these modern-day biomedicine tools is based on the encapsulation of senolytic drugs by galacto-oligosaccharide-coated silica porous scaffold nanoparticles (GalNP). Functionalized GalNP carriers can successfully transport and deliver small molecules such as doxorubicin and navitoclax to CDK4/6 inhibitor palbociclib-induced senescent lesions in vivo. Upon endocytosis and fusion with lysosomal vehicles, the cargoes are efficiently released by β-galactosidase-mediated hydrolysis, promoting GalNP-mediated senolysis and the subsequent regression of melanoma and lung squamous cell carcinoma tumor xenografts. Finally, using GalNPs for delivery effectively reduces the common cytotoxic effects of doxorubicin and navitoclax [218]. In essence, the synergistic treatment of senescence inducers and senolytic compounds is seemingly conceivable with such nanoparticle tools [219]. Additional proof-of-concept drug delivery studies confirm that nanocarriers entail a remarkable potential for senotherapies which can be further improved by changing their size and composition or tethering them to different polymers and epitopes (e.g., β2 microglobulin) or antibodies (e.g., CD9) against membrane markers in order to provide enhanced selectivity against senescent lesions [220,221,222,223,224].

Increased lysosomal content accompanied by elevated levels of SA-β-gal activity is a universal marker of senescent cells. Within this framework, several groups have recently opted to develop a new class of broad spectrum senolytic agents using this marker. In one of these strategies, galactose-modified duocarmycin (GMD) prodrugs are favorably processed in senescent cells by lysosomal β-galactosidase. Duocarmycin is a non-specific cytotoxic DNA-alkylating compound, however the enzymatic conversion of GMDs into duocarmycin drugs causes the specific destruction of senescent cells in vitro and in vivo, without affecting normal cells when applied at low doses. The study has also shown that GMD prodrugs can effectively eliminate chemotherapy-associated bystander senescent cells in preneoplastic lesions [225]. Gemcitabine-derivative SSK1 is another recently developed lysosomal SA-β-gal-responsive prodrug that selectively kills senescent cells in vitro and in vivo, independent of the senescence inducer [226].

## 6. Conclusions

Overall, cancer cell senescence is a Jekyll and Hyde phenomenon with both beneficial and detrimental implications. Despite a primary and immediate tumor-suppressive role against cancer development, the long-term consequences of senescent cancer cells are potentially deleterious. Recent advances in senotherapeutic strategies targeting senescent cells and their SASP has expanded the field of translational research on cancer therapies. The series of basic and translational studies presented in this review is exceptionally promising yet somewhat challenging to implement. This is partly because the pathways being targeted by existing senotherapies function in non-senescent cells, and their high dose and long-term use may cause adverse effects on other cell-types and tissues. Thus, safety issues pose a major concern, especially when the drugs are administered systemically. There are several possible ways to obtain higher efficacy and better safety profiles, such as focusing on the spatiotemporal optimization of treatments and using less toxic doses of senotherapeutic drugs by developing combination therapies and senescent cell-specific delivery systems. Another limitation with the current studies is that the experimental models may not perfectly recapitulate the heterogeneous and complex nature of disease conditions specific to human pathophysiology. Nonetheless, we envision that a comprehensive understanding of the cellular senescence mechanisms and careful evaluation of the heterogeneous nature of senescent cancer cell populations will help better translate these findings into clinical settings.
